# Attitudes toward evidence-based practices, occupational stress and work-related social support among health care providers in China: A SEM analysis

**DOI:** 10.1371/journal.pone.0202166

**Published:** 2018-08-10

**Authors:** Shan Qiao, Xiaoming Li, Yuejiao Zhou, Zhiyong Shen, Bonita Stanton

**Affiliations:** 1 Department of Health Promotion, Education, and Behavior, South Carolina SmartState Center for Healthcare Quality (CHQ), University of South Carolina Arnold School of Public Health, Columbia, South Carolina, United States of America; 2 Guangxi Center of Disease Control and Prevention, Nanning, Guangxi, China; 3 Hackensack-Meridian School of Medicine, Seton Hall University, South Orange, New Jersey, United States of America; University of Illinois at Chicago, UNITED STATES

## Abstract

Individuals’ attitudes toward evidence-based practices (EBP) are critical in adopting, implementing and maintaining the EBP in clinical settings. Multiple empirical studies have examined how work context may shape perceptions and attitudes towards EBP. The current study aims to further explore how both work and family contexts, as assessed by three psychosocial indicators (i.e., occupational stress, work-related social support from coworkers, and work-related social support from family), may affect attitudes toward EBP among health care providers in HIV clinics in China. We analyzed cross-sectional survey data from 357 health care providers recruited from 40 HIV clinics across 16 cities/counties in Guangxi China. Structural equation model (SEM) was constructed to test the hypothesized relationships among key study variables. Occupational stress was negatively associated with work-related social support from coworkers (β = -.19, 95%CI = [-.31,-.12]), which in turn was positively associated with attitudes toward EBP (β = .17, 95%CI = [.04, .30]). Similarly, occupational stress was negatively related to work-related social support from family (β = -.34, 95%CI = [-.42,-.25]), which in turn was positively related to attitudes toward EBP (β = .23, 95%CI = [.12, .35]). Occupational stress was negatively associated with attitudes toward EBP, but the magnitude of association did not reach statistical significance at α = .05. Work-related social support from family partially mediated the association between occupational stress and attitudes toward EBP (Sobel’s z = 3.27, p < .05). Our findings suggest the importance of integrating work and family contexts, especially family support into the strategies of facilitating the adoption and implementation of EBP. The current study also underscores the needs to reduce occupational stress and enhance work-related social support among health care providers who are in frequent contact with HIV patients. In addition, lack of work-related family support may be a main barrier preventing health care providers from developing a positive attitude toward EBP. Therefore, the interventions aiming for promoting adoption and utilization of EBP need to involve specific strategies to resolve work-family conflicts and improve family members’ understanding and support for health care providers in China, especially those who work in a stressful work context such as HIV care.

## Introduction

Implementation science literature has suggested that individuals’ attitudes always play a critical role in adopting, implementing, and maintaining evidence-based practices (EBP) in clinical settings [1, 2]. Individuals’ overall positive or negative attitudes and judgement toward the EBP may directly affect their behaviors of either supporting or resisting the implementation of the EBP [3].

Multiple empirical studies have examined how individuals’ attitudes may be impacted by work contexts. Fuller and colleagues reported that peer influence and organizational stress were associated with individuals’ attitudes toward EBP among the program administrators and staff in the National Drug Abuse Treatment Clinical Trials Network [4]. A study based on 303 mental health service clinicians indicated that both transformational and transactional leadership were positively associated with attitudes toward adoption of EBP among providers [5]. A recent study based on a nationwide sample of 1,112 health care providers from 100 mental health clinics suggested that more proficient organizational cultures and less stressful organizational climates were related to clinicians’ positive attitudes toward EBP adoption [6].

Although existing literature has accumulated considerable theoretical and empirical knowledge about individuals’ attitudes toward EBP in clinical settings, there are several research gaps. First, limited empirical studies have examined how both work and family contexts may shape perceptions and attitudes towards EBP. Second, although existing studies have identified specific factors that may influence individuals’ attitudes toward EBP, the mechanisms underlying these relationships and how such factors may interact with each other remain unclear. Third, almost all existing studies have been conducted in North America, and thus findings may have limited generalizability to other cultural settings, especially resource-limited settings. The current study aims to explore how both work and family contexts, assessed by three psychosocial indicators (i.e., occupational stress, work-related social support from coworkers, and work-related social support from family), may affect attitudes toward EBP among health care providers in HIV clinics in China.

Following the conceptual framework by Aarons (5), we examined four domains of individuals’ attitudes toward EBP in the current study: appeal of the EBP, requirements to adopt the EBP, openness to EBP in general, and perceived divergence between current work processes and those required by the EBP. The individuals’ attitudes can be measured by the willingness to adopt the EBP when it is used correctly or employed by colleagues (“appeal”), or the EBP is required by an agency (“requirement”). The individuals’ attitudes toward the EBP also depend on the extent to which the individuals are open to EBP and willing to try the EBP (“openness”); and how much the individuals agree that the EBP is useful in clinical settings and the EBP can play a more important role in daily practice compared with his/her existing clinical experience (“divergence”).

We also examined these domains in the contexts of work-related stress and social support available to the health care providers. Stress refers to physiological responses and behavioral tendencies that occur in response to a crisis or imbalance between demands and resources [7]. Occupational stress is considered to result from exposure to stressors at workplace. Health care providers may suffer occupational stress caused by imbalance of work demand, skills, supportive resources, and organizational factors (e, g., leadership, reward-system) [8–10]. Health care providers in HIV clinics may face additional stressors including stigma and discrimination associated with caring for HIV patients and the risk of occupational exposure [11]. Occupational stress may lead to mental health problems (e.g., depression, anxiety, and burnout)[12–14], physical health problems (e.g., sleep disorder, cardiovascular disease, and low immunity)[15–17] as well as negative organizational impacts (e.g., job dissatisfaction, decreased work performance, and deteriorated quality of service provision) [18, 19].

Social support refers to the perceived available assistance and the actual received assistance from others [20]. It typically consists of information support, material support, instrumental support, and emotional support [21]. Social support for daily work and career development (“work-related social support”), as an important type of resource for employees, may affect their perception of work-related stressors and their ability to cope with occupational stress [19, 22]. Health care providers who receive more social support view work-related stress as less threatening and may be able to cope more positively and effectively [23]. For example, social support at work among nurses was found to be positively associated with staying in their job, professional development, satisfaction with their work, and negatively associated with stress reactions, burnout, and absenteeism [20]. Work-related social support may also mediate the effects of occupational stress on depressive symptoms among health care providers [24], promote work engagement [25], and facilitate effective commitment to the organization [26]. Work-related social support can be obtained from various sources including supervisors, colleagues, family members, and friends [27–29]. Although a number of studies have demonstrated the role of social support in job satisfaction [30], work-family conflict [29] and mental health [28, 31], there is a dearth of empirical studies that explore the relationship among work-related social support, occupational stress and individuals’ attitudes toward EBP [32].

**Fig 1:**
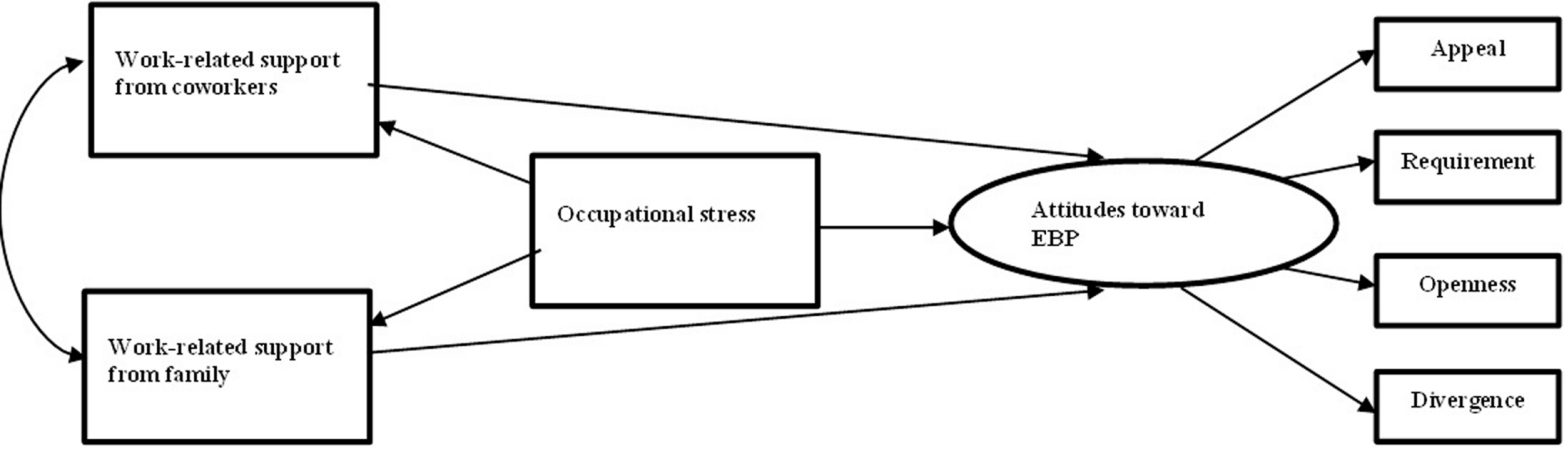
Conceptual framework.

Individuals’ attitudes function in and are shaped by a complex context including both workplace and family [33, 34]. We hypothesize that work and family contexts will influence individuals’ attitudes towards EBP. Specifically, we hypothesize that 1) occupational stress is negatively related to attitudes toward EBP; 2) a higher level of work-related social support from both coworkers and family is associated with a more positive attitude toward EBP; 3) work-related social support from both coworkers and family mediates the relationship between occupational stress and individuals’ attitudes toward EBP. The conceptual framework of this study is illustrated in Fig 1.

## Methods

### Study site

As of the end of September 2017, there were 747,000 people living with HIV (PLWH) in China [35] . Guangxi has experienced a fast growing HIV epidemic and currently ranks second among 31 Chinese provinces in terms of HIV seropositive cases [36]. Guangxi Center of Disease Control and Prevention (Guangxi CDC) has reported a total of 80,480 HIV/AIDS cases by October 2017, representing a 15.7% increase since June 2011 (69,548 HIV/AIDS cases)[36].

The current study was embedded in an existing NIH-funded HIV disclosure intervention trial for parents living with HIV in Guangxi, China. As an innovative intervention in HIV care practice, the intervention, entitled “Interactive Communication with Openness, Passion, and Empowerment” (ICOPE), aims to assist HIV-infected parents in making a planned and developmentally appropriate disclosure of their HIV infection to their seronegative children aged 6–15 years. The ICOPE trial consisted of intervention modules for both HIV-infected parents and health care providers working in HIV care. Data used in the current study were derived from the baseline survey among health care providers who participated in the ICOPE trial.

### Sampling

In Guangxi, each urban district and rural township has one designated primary public hospital (specifically its HIV clinic) that is responsible for clinical management and semi-annual follow-ups for all HIV/AIDS patients in the district or township. The disclosure intervention was conducted in six cities and ten rural counties with the largest number of reported HIV/AIDS cases by 2012. In collaboration with Guangxi CDC, we identified all the HIV clinics with at least 200 HIV/AIDS cases in both urban districts and rural townships and randomly selected 40 of them to participate in the ICOPE trial.

### Participants and survey process

All the health care providers (e.g., physicians, nurses, and case managers) working in the 40 HIV clinics across the 16 cities/counties were asked to participate in the baseline survey. In total, 357 health care providers completed a self-administered survey including measures on background characteristics (e.g., demographic and work-related characteristics), occupational stress, work-related social support from the family and coworkers, and individuals’ attitudes toward EBP. All participants provided written informed consent. The survey took 15–20 minutes on average to complete. The study protocol including consenting process was approved by the Institutional Review Boards at Guangxi CDC in China and at Wayne State University in the United States.

### Translation of the measurement scales

Some measurement scales were available only in English and then were translated into Chinese. Following an established protocol in previous research [37], the translation was initially performed by English-Chinese bilingual research team members. The Chinese translation was reviewed by local research staff in Guangxi CDC to check its cultural appropriateness for health care practice in China and was finalized based on their feedback. All measures were then translated back into English to examine if the meanings of the items had changed or lost in the translation process. Items that appeared to have changed in meaning were adjusted until the meaning met with the research staff’s intentions. Finally, the translated scales, along with other scales in the survey, were pilot tested in HIV clinics that were not part of the final intervention trial. The survey questionnaire was modified and finalized based on the feedback from the participants and local research staff.

## Measurement

### Background characteristics

Participants provided background information including demographic and work-related characteristics. Specifically, they were asked about gender, age, ethnicity, marital status, and educational attainment. They also reported professional rank, job title, years engaged in health care, years engaged in HIV prevention and care, whether they had received HIV-related training in the past year, and frequency of HIV patient contacts during work.

### Individuals’ attitudes toward EBP

The individuals’ attitudes toward EBP were assessed by the Evidence-based Practice Attitude Scale (EBPAS)[5]. This 15-item scale consists of four subscales including appeal, requirements, openness, and divergence. The Appeal Subscale assesses the extent to which the respondent would adopt an EBP (e.g., “if you received training in a therapy or intervention that was new to you, how likely would you be to adopt it if it was intuitively appealing”). The Requirements Subscale assesses the extent to which the respondent would adopt an EBP if “it were required by an agency, supervisor, or state”. The item of requirement by the state was removed based on the suggestions from the local research team as they anticipated very little variation in responses to this item (The medical practice was largely governed by the health bureaus. Health care providers must comply with what the government requires). The Openness Subscale assesses the extent to which the respondent likes to use EBP (e.g., “I like to use new types of therapy/ interventions to help my clients”). The Divergence Subscale assesses the extent to which the respondent perceives EBPs as not “clinically useful” and less important than clinical experience (e.g., “I know better than academic researchers how to care for my clients”). Each item of the EBPAS was originally rated on a 5-point scale ranging from 0 = “not at all”, 1 = “to a slight extent”, 2 = “to a moderate extent”, 3 = “to a great extent”, to 4 = “to a very great extent”. In the process of translation and pilot study, following the suggestions from local research team, the response “to a great extent” and “to a very great extent” were combined into one category because it was difficult to differentiate these two responses (“great” vs “very great”) in Chinese language. All items from the Divergence Subscale were reverse-coded in computing the overall EBPAS score. A higher overall EBPAS score indicates a more positive attitude toward EBP. The Cronbach α for the four subscales (Appeal, Requirements, Openness, and Divergence) were .78, .77, .90, and .50, respectively.

### Occupational stress

The Work-Related Strain Inventory (WRSI) [38] was adapted for the current study to measure occupational stress. The original 18-item WRSI covers common psychological problems or behaviors associated with feelings of strain in occupational setting (e.g., “It seems like I cannot get the recognition that I deserve”). It uses a 4-point response scale, ranging from 1 = “does not apply to me at all” to 4 = “always applies to me”. To shorten the scale, exploratory factor analysis was conducted with the original 18 items, and 14 items with factor loading ≥ .50 were retained for the current study. Some items were reverse-coded in computing the overall WRSI score. The total score is a global assessment of perceived occupational stress with a higher score suggesting a stronger feeling of work-related psychological strain. The Cronbach α was .77 for the current sample.

### Work-related social support from coworkers

The work organization exposure matrix (WOM) [39] has been used in a number of studies to assess psychosocial aspects of work context. This scale consists of 18 items covering three domains including psychological demands, decision latitude, and work-related social support from coworkers. The work-related social support subscale was used in the current study, work-related social support from coworkers was assessed by four items dealing with interaction with coworkers at work and outside work (e.g., “Can you talk to coworkers during breaks?”). The item responses consisted of a 4-point scale ranging from 1 = “not at all” to 4 = “to a very great extent”, with higher score indicating higher level of social support. The Cronbach α for the subscale was .72 for the current sample.

### Work-related social support from family

The Family Support Inventory for Workers [40] was adapted to measure the work-related social support from family. Following the suggestions from the local research team, twelve items of emotional support that were culturally appropriate for the local context were selected for use in the current study. The emotional support subscale includes behaviors and attitudes reflecting the family’s interest in the respondent’s work, willingness to listen to, talk to, and advise the respondent about his/her work, and general indications of care and concern for the respondent (e.g., “My family members have a positive attitude toward my work”) The 4-point response scale ranged from 1 = “strongly disagree” to 4 = “strongly agree”. After appropriately reverse scoring, the responses to the 12 items were summed with a higher total score indicating higher level of social support from family. The Cronbach α was .83 for the sample in our study.

### Data analysis

Descriptive analysis was employed to describe the participants’ background characteristics (e.g., basic demographic variables and work-related variables) and key study variables (i.e., attitudes toward EBP, occupational stress, work-related social support from coworkers and family). Gender difference in background characteristics and key study variables were compared using Chi-square tests for categorical variables and one-way ANOVA for continuous variables. We further examined if any background characteristics were associated with key study variables using one-way ANOVA (for the categorical background variables) and Pearson correlation coefficients (for continuous background variables). Pearson correlation coefficients were also calculated to examine the associations between key study variables. All statistics analyses were conducted with SPSS 24.0.

Structural equation model (SEM) was constructed to test the hypothesized relationships among key study variables as depicted in the conceptual framework (Fig 1). The SEM analysis was based on covariance matrix and performed with maximum likelihood method of parameter estimation using MPlus 6.0. Potential covariates (background variables) were also included in the final model to control for potential confounding effects of these variables. We first estimated standardized coefficients for all paths and evaluated goodness of model fit using χ^2^ statistics, Root Mean Square Error of Approximation (RMSEA), Bentler’s Comparative Fit Index (CFI), and Tucker-Lewis Fit Index (TLI) [41]. Models with a non-significant χ^2^ statistics, an RMSEA less than 0.08, and values of CFI and TLI larger than 0.90 were viewed as acceptable in terms of model fit [41, 42].

Considering the potential violation of multivariate normality in data, Satorrs-Bentler χ^2^ statistics was estimated as a scaling correction for χ^2^ statistics. The Satorrs-Bentler χ^2^ statistics incorporates model construction, parameter estimation method, and kutoisis values and has been shown to be a reliable test statistic for evaluating covariance structure models under various distribution and sample sizes [43, 44]. We obtained and examined χ^2^ statistics, Satorrs-Bentler χ^2^ statistics, CFI, TLI, and RMSEA.

To test whether the effect of occupational stress on attitudes toward EBP was significantly reduced upon the inclusion of potential mediators (i.e., work-related social support from coworkers and family) in the model, we calculated the Sobel’s Z statistic, a commonly used statistic for testing the significance of mediation effect [45].

## Results

### Background characteristics

As Table 1 shows, 55% of the participants were female and 45% were of Han ethnicity. The mean age of the sample was 35.0 years (SD = 8.65). Nearly 72% of the participants were married. About 55% of the participants had post-secondary degrees, and 25% of the participants graduated from universities. In term of professional rank, 31% of the participants were at entry level, 43% were at junior level, 25% were at intermediate level, and less than 2% at senior level. Most (77%) of the participants had no administrative title, nearly 22% worked as the header of their department and less than 2% were managers of their HIV clinics. On average, participants had worked in health care field for 12.5 years (SD = 9.07) and had engaged in HIV-related work for 4 years (SD = 3.90). Among the participants, over 94% had received HIV-related training, and 73% received such training in past year. Around 37% of the participants reported that they often had contact with HIV patients, and 19% reported contact with HIV patients on a daily basis.

**Table 1:** Background characteristics by gender.

N(%)	Overall (n = 357)	Male (n = 161, 45.1%)	Female (n = 196, 54.9%)
***Demographic variables***			
**Age (Mean, SD)**	34.98 (8.65)	35.63(8.99)	34.42(8.32)
**Ethnicity**			
Han	222 (65.9%)	121 (77.1%)	101(56.1%)
Non-Han	115 (34.1%)	36 (22.9%)	79(43.9%)***
**Marriage status**			
Married	244(72.4%)	119 (75.8%)	125 (69.4%)
Non-married	93(27.6%)	38 (24.2%)	55 (30.6%)
**Education**			
Junior middle school	30(8.9%)	11(7.0%)	19(10.6%)
Senior middle school	36(10.7%)	14(8.9%)	22(12.3%)
Post-secondary degrees	185(55.1%)	90(57.3%)	95(53.1%)
University	85(25.3%)	42(26.8%)	43(24.0%)
***Work-related variables***			
**Professional rank**			
Entry level	109(30.6%)	61(38.1%)	48(24.5%)
Junior	154(43.3%)	61(38.1%)	93(47.4%)
Intermediate	88(24.7%)	36(22.5%)	52(26.5%)
Vice senior	5(1.4%)	2(1.3)	3(1.5%) *
**Administrative title**			
None	246 (76.6%)	97(69.3%)	149(82.3%)
Department heads	69(21.5%)	40(28.6%)	29(16.0%)
Clinic leader	6(1.9%)	3(2.1%)	3 (1.7%)*
**Received HIV-related training before**			
Yes	335(93.8%)	151(93.8%)	184(93.9%)
**Received HIV-related training last year**			
Yes	260(73.4%)	134(83.2%)	126(65.3%)***
**Frequency of HIV patient contacts**			
None	25 (7.0%)	5(3.0%)	20(10.3%)
Sometimes	129(36.2%)	60(37.3%)	69(35.4%)
Often	133(37.4%)	73(45.3%)	60(30.8%)
Everyday	69(19.4%)	23(14.3%)	46(23.6%)**
**Years of working in health care area (Mean, SD)**	12.50 (9.07)	12.44 (9.31)	12.55 (8.89)
**Years of working in HIV-related area (Mean, SD)**	4.15 (3.90)	4.15 (3.62)	4.15 (4.11)

*p<0.05;

**p<0.01;

***P<0.001.

Generally, there was no significant gender difference in most background characteristics with a few exceptions. The proportion of Han ethnicity was higher among male participants than females (77% vs. 56%, *p* < .001). Compared to females, a higher proportion of male health care providers were at low professional rank (e.g., entry level: 38% vs. 25%; junior level: 38% vs. 47%; intermediate level and above: 24% vs. 28%; *p* = .021). In addition, a higher proportion of male health care providers reported contacting HIV patients often or everyday than the female providers (60% vs. 54%, *p* = .014).

### Associations between background characteristics and key study variables

The associations between categorical variables and key study variables are demonstrated in Table 2. Female providers scored higher on work-related social support from family than males (F = 10.44, *p* = .001). Health care providers’ education attainment was positively associated with work-related social support from coworkers (F = 3.51, *p* = .016). In addition, higher professional rank was significantly related to more work-related social support from coworkers (F = 3.94, *p* = .009). It is notable that higher frequency of HIV patient contacts was associated with higher level of occupational stress (F = 2.83, *p* = .038) and lower level of work-related social support from family (F = 2.94, *p* = .034). Attitudes toward EBP did not differ by any categorical background characteristics.

**Table 2:** ANOVA results of key study variables by background characteristics (categorical variables).

		Gender	Ethnicity	Marriage status	Education attainment	Professional Rank	Administrative title	Training before	Training last year	Freq. of HIV patient contacts
Attitudes toward EBP	F	.940	.013	.028	.708	1.530	.030	1.375	.079	1.038
Occupational stress	F	2.549	.066	.629	1.066	.820	1.366	.026	3.217	2.833*
Work-related support from coworkers	F	.015	.242	.383	3.507*	3.944**	.788	.621	3.039	1.707
Work-related support from family	F	10.438**	.042	.258	1.242	1.429	.430	.421	.625	2.935*

*p<0.05;

**p<0.01.

Table 3 shows the Pearson correlation coefficients between continuous variables and key study variables. Health care providers’ age was negatively associated with work-related social support from coworkers (r = -.172, *p* = .002). In addition, the longer they had worked in health care field, the lower level of work-related social support they received (r = -.141, *p* = .011for co-worker support; r = -.160, *p* = .004 for family support). More years engaging in HIV-related work was negatively associated with occupational stress (r = -.150, *p* = .007). There were no significant associations between attitudes toward EBP and any continuous background variables.

**Table 3:** Correlations (Pearson’s r) between key study variables and background characteristics (continuous variables).

	Age	Years working in health care field	Years working in HIV field
Attitudes toward EBP	-.006	-.021	.048
Occupational Stress	-.041	.061	-.150**
Work-related support from coworkers	-.172**	-.141*	.006
Work-related support from family	-.093	-.160**	.046

*p<0.05;

**p<0.01.

### Correlations among key study variables

The correlation matrix of the key study variables is shown in Table 4. All variables were significantly correlated with each other. Specifically, attitudes toward EBP was negatively related to occupational stress, but positively associated with work-related social support from both coworkers and family. Higher level of occupational stress was related to lower level of work-related social support. Work-related social support from coworkers was positively associated with the one from family.

**Table 4:** Correlation coefficients of key study variables.

	1	2	3	4
1. Attitudes toward EBP	1			
2.Occupational Stress	-.174**	1		
3.Support from coworkers	.140*	-.314***	1	
4.Support from family	.250***	-.397***	.295***	1
Mean	2.8	2.1	3.0	2.9
Standardized deviation	.37	.37	.42	.41

*p<0.05;

**p<0.01;

***P<0.001.

### Structural equation modeling

**Fig 2:**
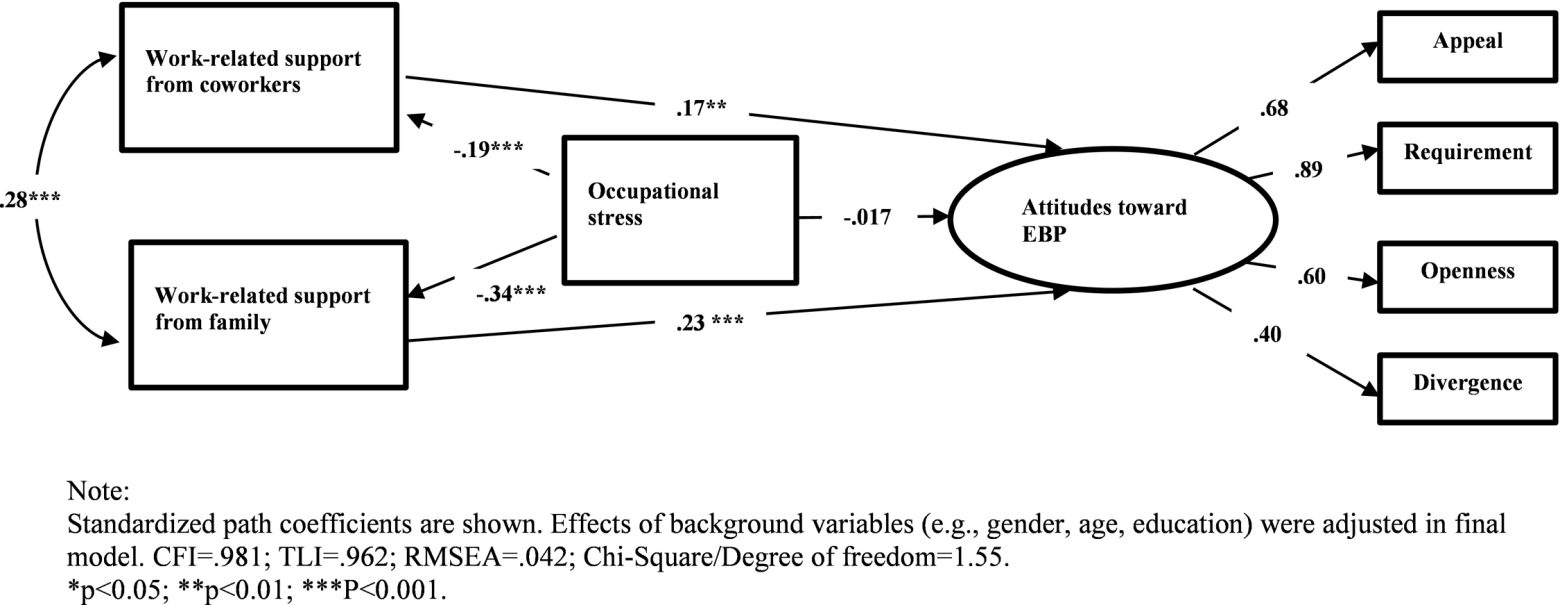
Final structural equation model.

In the SEM analysis, covariance coverage values (i.e., the proportion of data present to estimate each pairwise relationship) ranged from 99.7% to 100.0%. The background variables (e.g., gender, age, education) that are associated with key variables have been controlled in the final model. The final model with standardized path coefficients is illustrated in Fig 2.This model included five paths between the key study variables and fitted the data adequately (χ^2^ [14] = 21.746, *p* = .084; RMSEA = .042, 90%CI [.0001, .074]; CFI = .981; TLI = .962). Applying Satorrs-Bentler χ^2^ statistics, we obtained similar values for RMSEA, CFI, and TLI.

The standardized coefficients for the five paths in the model illustrate the associations among the key study variables. Occupational stress was negatively associated with work-related social support from coworkers (β = -.19, 95%CI = [-.31,-.12]), which in turn was positively associated with attitudes toward EBP (β = .17, 95%CI = [.04, .30]). Similarly, occupational stress was negatively related to work-related social support from family (β = -.34, 95%CI = [-.42,-.25]), which in turn was positively related to attitudes toward EBP (β = .23, 95%CI = [.12, .35]). Work-related social support might mediate the effect of occupational stress on attitudes toward EBP. Occupational stress was negatively associated with attitudes toward EBP, but the magnitude of association did not reach statistical significance at α = .05. Sobel z tests for the mediation effects suggested that work-related social support from coworkers did not mediate the association between occupational stress and attitudes toward EBP (Sobel’s z = 1.87, *p* = .06); work-related social support from family partially mediated the association between occupational stress and attitudes toward EBP given the effect of occupational stress was reduced to a non-significant level upon the inclusion of work-related social support from family to the model (Sobel’s z = 3.27, *p* = .001).

## Discussion

Adoption of EBP is a complicated process [46, 47]. Positive attitudes towards EBP could be a critical step in this process [48]. According to the Theory of Planned Behavior, beliefs, attitudes, and social norms will influence individuals’ intentions to engage in selected behaviors [49]. Intentions (e.g., self-instructions and motivations) to engage in innovative behaviors will lead to adoption and use of EBP [50]. A growing literature has addressed the importance of attitudes toward EBP in health care settings and explored factors at multiple ecological levels that may influence attitudes toward EBP [51, 52]. The current study explores how work and family contexts may influence individuals’ attitudes toward EBP. Our findings suggest that work-related social support from the family promotes attitudes favoring EBP, and mitigates the negative impact of occupational stress on attitudes toward EBP among health care providers in HIV clinics.

One recent meta-analysis of predictors of individuals’ attitudes toward EBP found that work context for EBP was positively related to individuals’ attitudes toward EBP, while general positive working climate was even more strongly related to individual innovative performance [53]. Occupational stress may hinder the implementation of EBP, as those have stress may perceive the work environment to be negative and experience organizational conflicts (e.g., unfairness, lack of reward, large workload, interpersonal tensions) [18, 54]. Occupational stress may also weaken the effect of positive working climate on individuals’ attitudes toward EBP [32]. Our study provides new empirical evidence on the negative impact of occupational stress on implementation of EBP and shows the importance of creating positive working context and reducing work-related stress in health care settings.

Social support has been identified as a critical factor in adopting and implementing EBP [55, 56]. Many empirical studies focus on the positive role of social support from supervisors, coworkers, or technical and administrational department within the organization in implementation of some EBP [57, 58]. Although a dearth of literature explores the impact of work-related social support from coworkers and family on adoption and implementation of EBP, a number of empirical studies indicate that work-related social support may reduce occupational stress level [19, 23]. In addition, social support from coworkers may modify the association between occupational stress and negative psychological condition [28] and relieve the work-family conflict among health care providers [59, 60]. Work-related social support from coworkers and family may contribute to creating psychologically positive work and family contexts. The perceived positive contexts may facilitate the implementation of EBP in health care settings [61]. The current study indicates that social support may buffer the negative impacts of occupational stress on the adoption of EBP. It also suggests that family may play an important role in facilitating implementation of EBP in clinical settings.

The current study did not find any significant associations between individual level factors (e.g., demographic and work-related characteristics) and attitudes toward EBP. However, a national study in the United States suggested that years of working experience and ethnicity were significantly related to the adoption of EBP among mental health clinicians [6]. Another study among child welfare workers indicated that buy-in for an organizational EBP in child welfare was related to gender and years of service. Male staff and staff with 16 or more years of service in the agency reported greater buy-in [54]. The mixed results regarding the individual level factors may attribute to different social contexts and various health care fields across studies.

The findings on the associations between background characteristics and the occupational stress and work-related social support will inform the selection of potential targets for future interventions in EBP adoption and implementation. For example, health care providers in China with lower education attainment and lower professional rank might obtain less work-related social support from coworkers, and thus can be one of the prime targets of intervention. Similarly, health care providers who work on the front-line and frequently contact HIV patients appeared to experience higher levels of occupational stress and received less work-related social support from their family. They may therefore be a highly vulnerable group likely to experience working stress and work-family conflicts, and thus also may hold negative attitudes toward EBP. Targeted interventions toward those health care providers may be effective strategy in promoting the adoption and implementation of EBP in clinical settings.

Several limitations of the current study should be noted. First, the study has limitations in its data. We used cross-sectional data in the analysis, which limits our ability to investigate the causal relationships among the key study variables. Longitudinal study data and cross-lagged modeling analysis are recommended to further elucidate complicated interactions among social support, occupational stress and attitudes towards adoption of EBP. In addition, self-reported data through a survey are subject to common method bias and other potential bias including social desirability [62].

Second, the study is subject to the limitations in measures. For example, we did not obtain other reliability statistics in addition of Cronbach α for the scales in the current study. Evidence of validity was also not available for some of scales. Attentions are particularly needed to the measurement of attitudes toward EBP (i.e., the EBPAS). The response option of the EBPAS was reduced to 4- point scale from the original 5-point scale. While the modification was justified based on local cultural context, the impact of such deviation from the original measure is unknown. In addition, the Cronbach α for the Divergence Subscale of the EBPAS was low. The EBPAS has been recently expanded to 36-item version with 12 domains [63]. Further studies should be conducted to examine the measures of divergence in Chinese culture and also determine whether the new EBPAS domains may be related to organizational and individual characteristics of health care workers in China.

Third, the present analysis did not control for organizational factors. Although the size and organizational structure were similar across the HIV clinics participating in the study, the organizational climate for EBP and organizational cultures within these clinics might vary. Organizational context is another critical factor influencing the health care providers’ attitudes and adoption of EBP [6]. Therefore, a more comprehensive model may be established and tested in the future to depict interactions among factors at individual level, organizational level and environment level (work and family contexts) and their impacts on individuals’ attitudes toward EBP.

Fourth, we need to be cautious in generalizing the findings to other culture context. The current study was conducted in China where family is traditionally viewed as one of core values in its culture [64]. The cultural traditions in China may shape the contexts of health care practices and impact the interactions among occupational stress, work-related social support, and attitudes toward EBP.

## Conclusion

In spite of these limitations, our study is one of the first efforts to investigate how work and family contexts (indicated by occupational stress and work-related social support) may influence individuals’ attitudes toward EBP among health care providers in HIV clinics in China. The study may have some important implications for the implementation of evidence-based practices in clinic settings in China. For example, our findings suggest the importance of integrating daily environment, especially family support into the strategies of facilitating the adoption and implementation of EBP. The current study also underscores the needs to reduce occupational stress and enhance work-related social support among health care providers who are in frequent contacts with HIV patients. In addition, in a cultural context where attitudes of family members are usually respected and support from family members is valued [65], lack of work-related family support, may be a main barrier preventing health care providers from developing a positive attitude toward EBP. Therefore, the efforts to facilitate the adoption and implementation of EBP need to involve specific strategies to resolve work-family conflicts and improve family members’ understanding and support to the work of health care providers.
